# Direct Regulation of the T Cell Antigen Receptor's Activity by Cholesterol

**DOI:** 10.3389/fcell.2020.615996

**Published:** 2021-01-08

**Authors:** Salma Pathan-Chhatbar, Carina Drechsler, Kirsten Richter, Anna Morath, Wei Wu, Bo OuYang, Chenqi Xu, Wolfgang W. Schamel

**Affiliations:** ^1^Centre for Biological Signalling Studies and Centre for Integrative Biological Signalling Studies, University Freiburg, Freiburg, Germany; ^2^Department of Immunology, Faculty of Biology, University of Freiburg, Freiburg, Germany; ^3^Centre for Chronic Immunodeficiency (CCI), University of Freiburg, Freiburg, Germany; ^4^Immunology, Infectious Diseases and Ophthalmology Disease Translational Area, Roche Innovation Center Basel, Basel, Switzerland; ^5^State Key Laboratory of Molecular Biology, CAS Center for Excellence in Molecular Cell Science, Shanghai Institute of Biochemistry and Cell Biology, Chinese Academy of Sciences, Shanghai, China; ^6^School of Life Science and Technology, ShanghaiTech University, Shanghai, China

**Keywords:** cholesterol, lipid, TCR, signaling, T cell, nanocluster, allostery

## Abstract

Biological membranes consist of hundreds of different lipids that together with the embedded transmembrane (TM) proteins organize themselves into small nanodomains. In addition to this function of lipids, TM regions of proteins bind to lipids in a very specific manner, but the function of these TM region-lipid interactions is mostly unknown. In this review, we focus on the role of plasma membrane cholesterol, which directly binds to the αβ T cell antigen receptor (TCR), and has at least two opposing functions in αβ TCR activation. On the one hand, cholesterol binding to the TM domain of the TCRβ subunit keeps the TCR in an inactive, non-signaling conformation by stabilizing this conformation. This assures that the αβ T cell remains quiescent in the absence of antigenic peptide-MHC (the TCR's ligand) and decreases the sensitivity of the T cell toward stimulation. On the other hand, cholesterol binding to TCRβ leads to an increased formation of TCR nanoclusters, increasing the avidity of the TCRs toward the antigen, thus increasing the sensitivity of the αβ T cell. In mouse models, pharmacological increase of the cholesterol concentration in T cells caused an increase in TCR clustering, and thereby enhanced anti-tumor responses. In contrast, the γδ TCR does not bind to cholesterol and might be regulated in a different manner. The goal of this review is to put these seemingly controversial findings on the impact of cholesterol on the αβ TCR into perspective.

## Introduction

**Figure 1:**
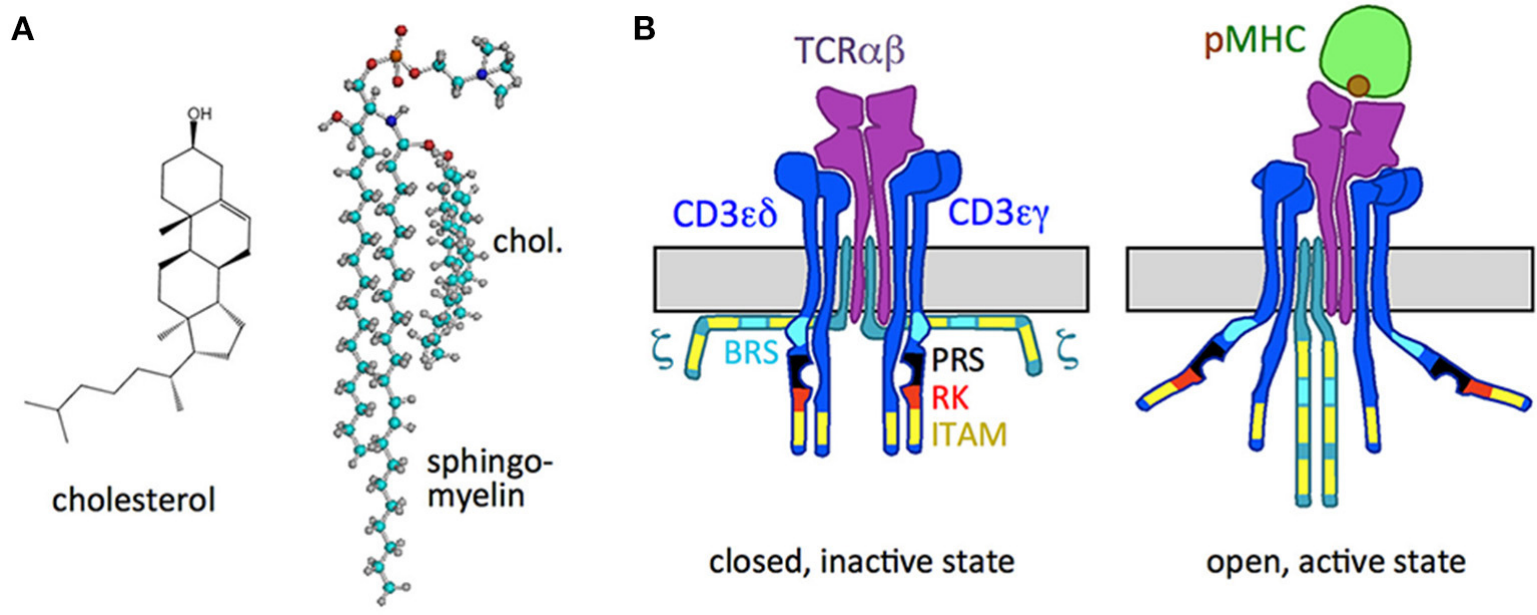
Cholesterol and the TCR. **(A)** Structure of cholesterol and the cholesterol sphingomyelin pair. **(B)** Schematic of the resting, *inactive* TCR, in which the cytoplasmic signaling motifs of the CD3 and ζ subunits are not accessible (right), and of the *active* TCR with the pMHC ligand bound (left), in which the motifs are exposed. The ITAM, BRS, PRS, and RK motifs are indicated.

A eukaryotic plasma membrane is composed of a variety of lipids and sterols, such as cholesterol. The most common composition of the plasma membrane is 20–50% phosphatidylcholine, 20–25% sphingomyelin, 30–50% cholesterol, 10% phosphatidylserine and 25% phosphatidylethanolamine (van Meer et al., 2008; Marquardt et al., 2015). One important sterol is cholesterol (Figure 1A), that is synthesized by the cells themselves and can be taken up from the environment. It determines membrane fluidity and permeability (Heerklotz and Tsamaloukas, 2006; Subczynski et al., 2017). The tetracyclic structure of cholesterol is planar and rigid. As a result, increase in membrane cholesterol increases lipid packing and stiffness causing decreased fluidity of lipid bilayers.

Lipids are not randomly distributed within the membrane but are organized. Using model membranes lipid nanodomains called liquid-ordered (Lo) and liquid-disordered (Ld) domains can be distinguished (Veiga et al., 2001; Veatch et al., 2004). It has been argued that these nanodomains are also present in the plasma membrane of living cells, although in a less stable and smaller manner (Eggeling et al., 2009; Levental et al., 2011; Mueller et al., 2011). The Lo domains would correspond to the lipid rafts in cellular membranes and the Ld domains to the non-raft domains (Simons and Ikonen, 1997; Sharma et al., 2004). In cellular membranes the lipid rafts are enriched in sphingolipids and cholesterol and are most likely very small (10–40 nm) and short-lived (microseconds) and hence difficult to characterize. Important for the formation of these domains is the interaction between cholesterol and sphingomyelin that facilitates stable dimers (Figure 1A) (Demel et al., 1977; Veiga et al., 2001; Bjorkbom et al., 2011). In addition to the dimer, free cholesterol and free sphingomyelin also exist (Simons and Ikonen, 1997; Endapally et al., 2019). Rafts concentrate signaling molecules and thus are important for signaling (Simons and Ikonen, 1997). Non-raft domains are rich in unsaturated glycerophospholipids, mostly lack sphingolipids and contain less cholesterol. Lo domains are thicker than Ld domains, due to the loss of kinks in acyl chains (Subczynski et al., 2017). Another factor that contributes to nanodomain formation in cellular membranes is the lipid asymmetry between the outer and the inner leaflet. For example, phosphatidylserine is strongly enriched in the inner leaflet and sphingomyelin is mainly found in the outer leaflet (Fadeel and Xue, 2009). Another well-known asymmetry observed is of that of cholesterol where its affinity toward sphingomyelin leads to its enrichment in the outer layer (Wood et al., 2011), although due to its small hydrophilic group (Figure 1A) it possesses a very high flip-flop rate (Steck et al., 2002).

Transmembrane (TM) proteins are also not randomly distributed on the cell surface, but localize to certain lipid nanodomains. This is most likely dictated by the exact sequence of the TM region that interacts with the lipids, but also by interactions with other proteins. This localization impacts the function of these proteins, as it allows the vicinity to proteins with a similar lipid preference and guarantees a distance to proteins with a different lipid preference. For example, specific interaction of TM proteins with certain lipids has been demonstrated by structural biology for the bacteriorhodopsin-glycolipid S-TGA-1 (Essen et al., 1998), the cytochrome bc1 complex of the mitochondrial respiratory chain (Hunte, 2005), the metarhodopsin-cholesterol (Ruprecht et al., 2004), the β2-adrenergic receptor-cholesterol (Cherezov et al., 2007; Hanson et al., 2008) interactions or by functional assays for the epidermal growth factor receptor (EGFR)-ganglioside GM3 association (Coskun et al., 2011). These interactions might be the underlying reason for their preferential localization to certain lipid domains or not. In addition, these specific TM region-lipid interactions might directly influence the function of the TM protein. One well-studied example is the T cell antigen receptor (TCR)-cholesterol interaction (Schamel et al., 2017, 2019) and this is the focus of this review.

## The T Cell Antigen Receptor (TCR)

T cells are important for an adaptive immune response against pathogens and tumors and are involved in autoimmunity. In humans 95% of the T cells express an αβ TCR while 5% express a γδ TCR on their surface. The TCR expression is crucial for their development and activation. The αβ TCR (here denoted as TCR for simplicity) binds to pathogen-, tumor- or host-derived peptides presented on MHC molecules (pMHC) by the host's cells. This binding leads to the activation and proliferation of the T cells and downstream effector functions such as cytokine production, provision of help to B cells, regulation of the T cell response or killing of cells expressing the cognate pMHC.

The TCR is a trans-membrane protein complex composed of non-covalently bound TCRαβ, CD3γε, CD3δε, and ζζ_2_ dimers (Figure 1B). All subunits are type I membrane proteins that contain either basic amino acid residues (arginine and lysine in TCRα; lysine in TCRβ) or acidic ones in their TM domains (aspartic acid in CD3ε, CD3δ, and ζ; glutamic acid in CD3γ) (Alarcon et al., 2003; Malissen, 2003). It is suggested that the potentially charged amino acids in the TM domains are involved in the interaction between the TCRαβ and CD3 (Call et al., 2002) as are also the ectodomains (Schamel et al., 2019).

With their variable extracellular regions TCRαβ bind to pMHC and the information of ligand binding is transduced through the membrane to the cytosolic tails of CD3 and ζ, that contain intracellular signaling motifs (Figure 1B). These motifs include the receptor-kinase (RK) motif that binds to the TCR's kinase Lck (Hartl et al., 2020), tyrosines in the context of the immunoreceptor tyrosine-based activation motifs (ITAMs) that can be phosphorylated (Reth, 1989; Weiss, 2010) and a proline-rich sequence (PRS) that can associate with the adaptor protein Nck (Gil et al., 2002). Further, basic rich sequences (BRSs) in CD3ε and ζ bind to negatively charged lipids of the inner leaflet of the plasma membrane in the resting TCR (Aivazian and Stern, 2000; Xu et al., 2008; Zhang et al., 2011). pMHC-binding leads to the exposure of these motifs with a consequent phosphorylation of the tyrosines by Lck. These phospho-tyrosines serve as docking sites for signaling proteins with SH2 domains (Acuto et al., 2008; Courtney et al., 2018). The latter then transduce the signal into the cells, causing activation of the T cell and subsequent effector functions.

## The αβ TCR Binds To Cholesterol

Compared to techniques to study protein-protein interactions, the ones for identifying specific associations of lipids with the TM regions of proteins are scarce and have limitations. Thus, not much is known about lipid-protein interactions. Useful techniques include the following: (i) In living cells covalent cross-linking of lipid derivatives with a UV light inducible reactive groups to proteins as been successfully used (Thiele et al., 2000; Hulce et al., 2013). However, the lipids are not exactly the natural ones and thus some interactions might not be detected. (ii) Structural studies of membrane proteins, such as NMR or crystallization, might resolve lipids that either were co-purified with the protein or added during the analysis or crystallization (Hunte, 2005). (iii) Although indirect, another approach is to modulate the lipid composition of the membrane, as e.g., is artificial liposomes, and then detect changes on the embedded membrane protein (Coskun et al., 2011). (iv) A complementary method is to use beads coupled to a lipid that are then used for pull-down assays to purify proteins that bind to the lipid (Beck-Garcia et al., 2013). However, this requires solubilisation of the membrane proteins by detergent that might be a source for artifacts. Due to these caveats it is recommended to use at least two complementary techniques. These experiments done with the TCR are described in the next paragraph.

Using a radioactive cross-linkable cholesterol derivative (Thiele et al., 2000), we could show that cholesterol specifically binds to the TCR in living cells, and it did not bind to other receptors tested (Molnar et al., 2012). This binding occurred to the TCRβ chain in the resting, i.e., non-ligand bound TCR (Molnar et al., 2012; Swamy et al., 2016). In a recent first high resolution structure of the complete TCR, bound cholesterol was not seen (Dong et al., 2019), most likely because digitonin was used to solubilize the TCR from the cell membrane, which is known to extract and remove cholesterol from the TCR (Schamel et al., 2005; Alarcon et al., 2006; Molnar et al., 2012). Interestingly, cholesterol sulfate, a naturally occurring derivative of cholesterol, competes with cholesterol in binding to the TCR (Wang et al., 2016).

The cholesterol-TCRβ interaction is dynamic, since only the non-ligand bound TCR associated with cholesterol and the ligand-bound TCR did not (Swamy et al., 2016). These binding characteristics were recapitulated using purified TCRs and cholesterol-coupled beads (Beck-Garcia et al., 2013; Swamy et al., 2016) as only the *resting* TCR bound to these beads. This demonstrated that the dynamic cholesterol binding is a property of the TCRβ TM region and is not a consequence of altered membrane properties caused by ligand engagement.

In addition to the cholesterol-TM region interaction and as mentioned above, the cytosolic tails of CD3ε and ζ might interact with the head groups of negatively charged lipids, such as phosphatidylserine, in the inner leaflet of the plasma membrane (Aivazian and Stern, 2000; Xu et al., 2008). Since this has already been reviewed by Wu et al. (2016), it will not be discussed.

## The γδ TCR Does Not Bind To Cholesterol

At first sight the γδ TCR looks similar to the αβ TCR. It also contains the CD3 and ζ subunits, but instead of TCRαβ it contains the highly related TCRγδ ligand-binding dimer. However, the TM region of TCRγ is partially different to the of TCRβ, and consequently using a radioactive cross-linkable cholesterol derivative (Thiele et al., 2000), we demonstrated that the γδ TCR does not bind to cholesterol (Swamy et al., 2016). Thus, the function of cholesterol on the activity of the TCR that we discuss in this review is limited to the αβ TCR and the γδ TCR must therefore be regulated by different mechanisms. A comparison of both TCRs was published recently (Morath and Schamel, 2020).

## Cholesterol Regulates The Allosteric Switch of The αβ TCR

As a prerequisite for allostery, the TCR exists in (at least) two different conformations that differ in their tertiary and/or quaternary structure (Figure 1B). Although most crystal structures of certain isolated domains of TCRαβ and CD3 did not provide information on these changes [Garboczi et al., 1996; Garcia et al., 1996; Rudolph et al., 2006; and the reason for that is discussed in a recent review Schamel et al. (2019)], a number of experiments have detected changes in the TCR structure upon ligand binding. These include NMR (Natarajan et al., 2017; Rangarajan et al., 2018), crystallography (Beddoe et al., 2009), and fluorescence-based or H/D exchange approaches (Beddoe et al., 2009; Hawse et al., 2012; Lee et al., 2015). In addition, biochemistry has provided evidence that the TCR structure changes when pMHC (or stimulating antibodies) are bound. These include limited trypsin digest (Risueno et al., 2008), measuring the distance between two subunits (Lee et al., 2015), accessibility of an antibody epitope (Risueno et al., 2005), cholesterol-binding to TCRβ (Swamy et al., 2016), and exposure of the proline-rich sequence (PRS) (Gil et al., 2002; Minguet et al., 2007), the receptor-kinase (RK) motif (Hartl et al., 2020) and the tyrosines in the cytosolic tails of the CD3 and ζ subunits (Swamy et al., 2016).

The two conformations of the TCR are: (i) the *resting, inactive* conformation (TCR), in which the CD3ε RK motif cannot bind to the Lck and the cytoplasmic tyrosines are shielded and thus are not phosphorylated; and (ii) the *active* conformation, which is stabilized after pMHC or antibody binding, in which Lck binds to CD3ε and the exposed cytosolic tyrosines of CD3 and ζ are phosphorylated (Gil et al., 2002, 2005; Minguet et al., 2007; Lee et al., 2015; Swamy et al., 2016; Hartl et al., 2020). The switch to the *active* conformation is essential for TCR phosphorylation and T cell stimulation. This was confirmed using artificial ligands (Minguet et al., 2007) and TCR mutants that are trapped in the *resting* conformation (Martinez-Martin et al., 2009; Blanco et al., 2014; Dopfer et al., 2014).

Thus, the αβ TCR is allosterically regulated; binding of pMHC at one site (through the variable regions of TCRαβ) causes structural alterations and dynamic changes at other sites, e.g., in the CD3 subunits. As a side note, the γδ TCR does not show these changes and its activity is regulated in a different manner (Blanco et al., 2014; Dopfer et al., 2014; Juraske et al., 2018; Morath and Schamel, 2020).

**Figure 2:**
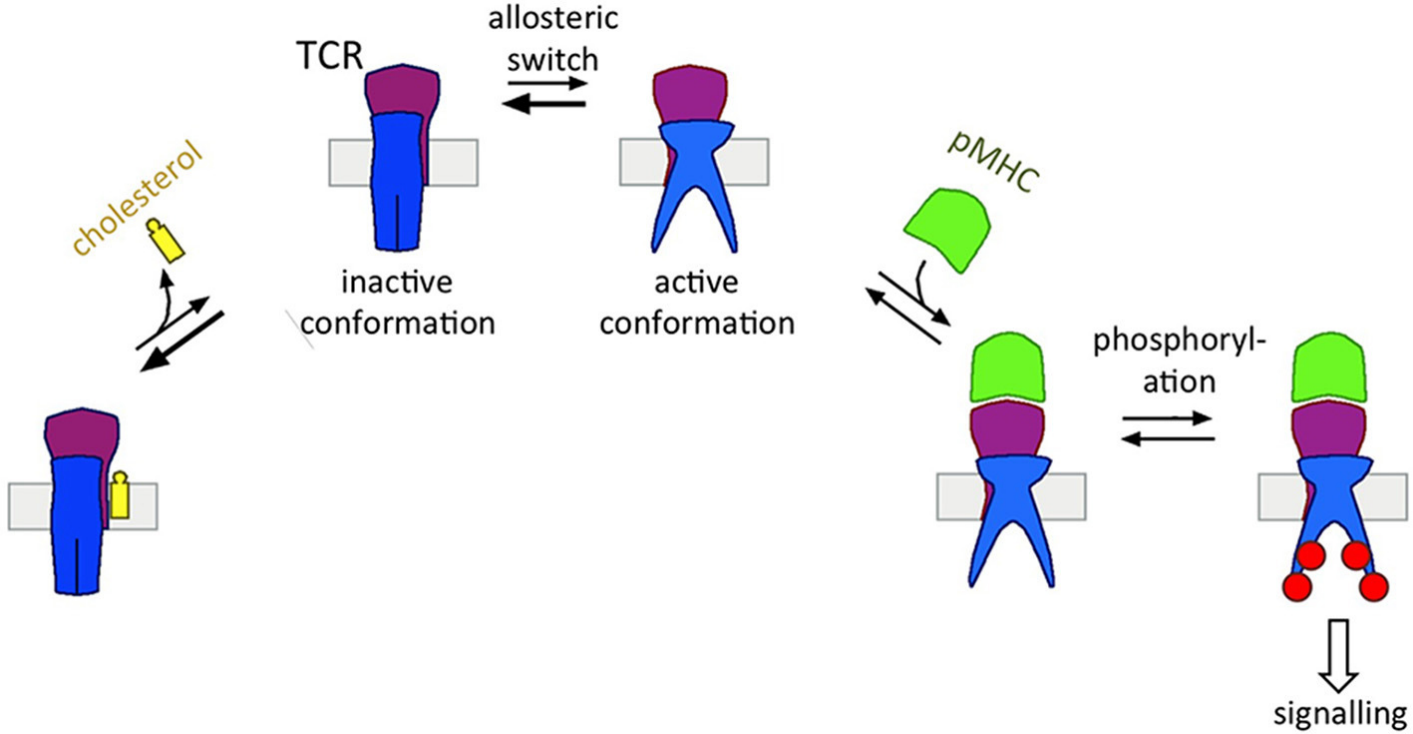
Cholesterol's function of regulating the allosteric switch of the TCR. The TCR can switch spontaneously between the *inactive* and *active* state (allosteric switch). Cholesterol binds to the TCR**β** subunit only in the *inactive* TCR, thus shifting the equilibrium to the left side. The pMHC ligand binds to the TCRα**β** subunits only in the *active* TCR, thus shifting the equilibrium to the right side. Only in the *active* state the TCR can be phosphorylated transmitting the signal of pMHC-binding downstream.

The Monod-Wyman-Changeux model of allostery (Monod et al., 1965) proposes that the αβ TCR can switch spontaneously between the two states in the absence of ligand (Figure 2) (Schamel et al., 2017); experimental evidences support this notion (Mingueneau et al., 2008; de la Cruz et al., 2011; Swamy et al., 2016). The ligand binding can perturb the equilibrium between these two states. In fact, ligand only binds to the *active* state and thus shifts the equilibrium to the *active* state (Swamy et al., 2016); consequently the cytoplasmic motifs are exposed and the TCR becomes signaling active (Figure 2). So how does a T cell guarantee that in the absence of ligand not too many TCRs are in the *active* state? This is achieved by cholesterol, which binds only to the *resting* TCR, and hence shifts the equilibrium to *inactive* TCRs (Figure 2) (Swamy et al., 2016). Thus, the TCR has two opposing binding partners, one that leads to the accumulation of *inactive* TCRs (cholesterol) and the other one that promotes *active* TCRs (pMHC).

The spontaneous shift of the TCR between its conformations was seen when in the absence of pMHC the cholesterol concentration was lowered (by extraction with methyl-β-cyclodextrin or by oxidation to cholestenone), which caused accumulation of *active* TCRs and initiated spontaneous TCR signaling (Kabouridis et al., 2000; Rouquette-Jazdanian et al., 2006; Swamy et al., 2016). Although methyl-β-cyclodextrin is commonly used to extract or to deliver cholesterol to membranes, it has several undefined effects on the plasma membrane and cell viability. Apart from increasing membrane permeability, it also depolymerizes the actin cytoskeleton and thereby reduces cell stiffness (Mundhara et al., 2019). Hence, it is important to complement the results obtained by methyl-β-cyclodextrin with other methods. In our previous studies we employed cholesterol oxidase to reduce the amount of available membrane cholesterol and again observed an accumulation of TCRs in the *active* state (Swamy et al., 2016). Similarly, mutating the TCRβ TM region so that cholesterol can no longer bind led to a shift of the equilibrium toward the *active* state and low level of T cell stimulation (Petersen et al., 2004; Swamy et al., 2016). These reports show that the TCR TM regions are key regulators of the conformational states of the TCR and that changes at the TM regions are linked to changes at the cytosolic tails.

In conclusion, cholesterol is a natural negative allosteric regulator of the TCR that guarantees that in the absence of ligand most TCRs remain in the *resting* state.

In another model, the TCR acts as a mechanosensor, in which force that is applied via pMHC to the TCR changes the TCR's structure to a signaling active configuration (Kim et al., 2009; Schamel et al., 2019). Since cholesterol stiffens the membrane, its presence at the TCR might influence these changes.

## Cholesterol Regulates αβ TCR Nanoclustering

**Figure 3:**
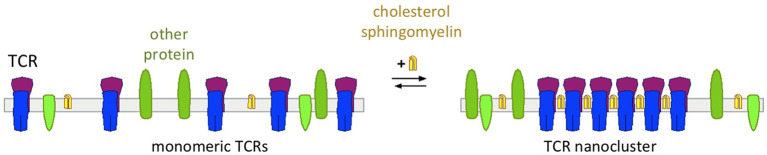
Cholesterol's function of regulating nanoclustering of the TCR. With low levels of cholesterol and sphingomyelin TCRs are expressed as monomers on the cell surface (left)—as it is the case in naïve T cells. With increasing concentrations of cholesterol and sphingomyelin, these lipids bind to the TCR and cause TCR nanoclustering—as it is the case in activated and memory T cells.

By using complementary techniques, several studies have suggested that on the surface of a resting T cell, the TCR exists in a monomeric and in a nanoclustered form (Schamel et al., 2005; Lillemeier et al., 2010; Kumar et al., 2011; Sherman et al., 2011; Schamel and Alarcon, 2013; Pageon et al., 2016; Martín-Leal et al., 2020). Other studies only found low amount of TCR nanoclusters and thus concluded that nanoclusters would not exist (James et al., 2011; Rossboth et al., 2018). Thus, the existence of TCR nanoclusters is still controversial, and technical limitations that contribute to this disagreement are discussed in several articles (Schamel and Alarcon, 2013; Platzer et al., 2020). For example, detergents might disrupt nanoclusters when being analyzed biochemically, in microscopy a low labeling efficiency might prevent the detection of nanoclusters or rapid blinking of a fluorophore attached to a TCR might lead to the detection of a nanocluster when in reality there is only one TCR present. Our own studies favor the existence of TCR nanoclusters. In fact, the amount and the size of the nanoclusters depend on the state of the cell (and this might be another confounding factor for detecting or not the nanoclusters). For example, a naïve T cell has less and smaller nanoclusters than an antigen-experienced T cell (Kumar et al., 2011; Schamel and Alarcon, 2013). Likewise, the cholesterol content of these cells increased from naïve to memory cells (Kaech et al., 2002; Kersh et al., 2003; Tani-ichi et al., 2005). These findings suggest that cholesterol is involved in the TCR nanoclustering (Figure 3) and three different approaches have shown that this is the case: (i) solubilisation of the TCR from T cell membranes with detergents that do not extract cholesterol preserved the TCR's nanoclustered form; in contrast, when cholesterol was extracted the nanoclusters disassemble to the monomeric TCRs (Schamel et al., 2005; Alarcon et al., 2006; Molnar et al., 2012). (ii) TCR nanoclusters disassembled when cholesterol was either extracted from the cells or when cholesterol levels were reduced pharmacologically as detected by immuno-gold electron microscopy or super-resolution fluorescence microscopy (Schamel et al., 2005; Alarcon et al., 2006; Molnar et al., 2012; Yang et al., 2016). (iii) Reconstituting the monomeric TCR in liposomes of defined lipid composition only allowed nanoclusters to form when cholesterol and sphingomyelin were present in the otherwise phosphatidylcholine-containing liposomes (Schamel et al., 2005; Alarcon et al., 2006; Molnar et al., 2012). This indicated that membrane proteins other than the TCR and lipids other than the ones mentioned are not required for TCR nanoclustering. Since sphingomyelin is mainly present in the outer leaflet of the plasma membrane, it is possible that cholesterol and sphingomyelin [maybe as a pair (Demel et al., 1977; Veiga et al., 2001; Bjorkbom et al., 2011)] bind to the N-terminal part of the TCRβ TM region. However, this remains to be tested. Concerning the mechanism of how cholesterol and sphingomyelin promote TCR nanoclustering, we suggested that cholesterol and sphingomyelin form a mini-raft-islet at the TCRβ TM region that is not favored to be in contact with the non-raft lipid domains that are around the TCR (Molnar et al., 2012; Beck-Garcia et al., 2015). Thus, these islets from several TCRs would come close to each other to shield each other from the non-raft domains, causing TCR nanoclustering.

By regulating TCR nanoclustering cholesterol defines the sensitivity of the TCR for activation through its ligand; a T cell with more and bigger nanoclusters is easier to activate than a cell with predominantly monomeric TCRs (Kumar et al., 2011). Indeed, it was shown that TCR nanoclusters possess a higher avidity toward multimeric pMHC than monomeric TCRs (Molnar et al., 2012). Further, TCRs within a nanocluster show positive cooperativity, so that if one TCR is stabilized in the *active* state by ligand-binding also the other TCRs in the nanocluster reside in the *active* state (Martinez-Martin et al., 2009; Schamel et al., 2017). TCRs in a given nanocluster that are stabilized in *inactive* state by cholesterol can spontaneously release cholesterol and thereby subsequently switch to the *active* conformation. Whether it is sufficient that one single TCR within a cluster binds to cholesterol to prevent the switch of all TCRs to the *active* conformation is not known. Since nanoclusters disassemble when cholesterol is extracted from the cells, the cooperativity of TCR within nanoclusters could be abrogated by cholesterol removal (Martin-Blanco et al., 2018). This result again showed that nanoclusters are required for the TCR cooperativity and that cholesterol is crucial for TCR nanocluster formation.

Studies show that naive T cells contain lower levels of cholesterol than activated T cells (Kersh et al., 2003; Tani-ichi et al., 2005). Indeed, upon activation of T cells cholesterol metabolism is reprogrammed to synthesize more cholesterol by upregulation of the Sterol Regulatory Element-Binding Protein-2 (SREBP-2) pathway and to transport less cholesterol out of the cell by downregulation of Liver X Receptor (LXR) target genes (Bensinger et al., 2008; Wu et al., 2016). Importantly, in antigen-experienced T cells, such as effector or memory T cells, the increased cholesterol levels contribute to enhanced TCR nanoclustering (Kumar et al., 2011). This might be a danger, since the nanoclusters lower the threshold for T cell activation due to increased avidity and cooperativity. Thus, a counter-regulation through cholesterol by keeping the TCRs in the *inactive* state might prevent spontaneous activation or activation by weak signals, in order to prevent autoimmune diseases. Indeed, elevated cholesterol levels in T cells have been linked to certain autoimmune diseases (see below).

Cholesterol sulfate, which is a low abundant derivative of cholesterol (Bergner and Shapiro, 1981) can bind to the TCR and disrupt the TCR-cholesterol interaction (Wang et al., 2016). This finding suggested that cholesterol sulfate binds to the same region as cholesterol. Interestingly, cholesterol sulfate disrupted TCR nanoclustering in liposomes and in T cells (Wang et al., 2016). The reduced TCR nanoclustering was paralleled by a reduced avidity of the T cells toward multivalent TCR ligands (Wang et al., 2016). Maybe the cholesterol-sphingomyelin pair is required for TCR nanoclustering (see above); and since cholesterol sulfate may not bind to sphingomyelin, it does not promote TCR nanoclustering.

Likewise, the lipid ceramide reduced TCR nanoclustering (Martín-Leal et al., 2020). This was observed in liposomes as well as in T cells that were treated with sphingomyelinase, which hydrolyses sphingomyelin to ceramide. This data suggest that the cholesterol-sphingomyelin pair drives TCR nanoclustering. Interestingly, signaling by the receptor CCR5 reduces ceramide levels in antigen-experienced T cells (Martín-Leal et al., 2020). In these cells, along with reduced ceramide levels, increased membrane cholesterol contributes to enhanced TCR nanocluster formation and increased sensitivity of these cells compared to naive T cells. In conclusion, T cells regulate their membrane lipid composition, in order to tune TCR nanoclustering and thus TCR signaling.

## Modulation of Cholesterol Levels To Tune αβ TCR Function In The Treatment of Diseases

As discussed, cholesterol modulates the activity of the TCR. Moreover, since dampening or increasing signaling by the TCR can be used to treat autoimmunity or cancer, respectively, it is not surprising that pharmacologically changing the cholesterol content of T cells has been used to ameliorate certain diseases.

Autoantibodies and the deposition of immune complexes are known to cause the autoimmune disease systemic lupus erythematosus (SLE). Overactive T cells contribute to the pathology by help provided to B cells and by the killing of host cells in a number of organs. Thus, a strong T cell activity contributes to SLE (Moulton and Tsokos, 2015). Importantly, T cells from SLE patients possess increased plasma membrane levels of cholesterol and glycosphingolipids (Jury et al., 2004; McDonald et al., 2014). These could lead to enhanced TCR nanoclustering and formation of signaling-promoting lipid rafts, consequently leading to increased T cell activation and effector functions as observed experimentally (McDonald et al., 2014). Extraction of cholesterol from the membrane of T cells from SLE patients using methyl-β-cyclodextrin indeed reversed the heightened signaling by the TCR (Krishnan et al., 2004). This may be a result of a disintegration of TCR nanoclusters and a partial disruption of lipid rafts. Reduced TCR signaling was also seen when the inhibitor N-butyldeoxynojirimycin was used, which normalized glycosphingolipid levels in T cells of SLE patients (McDonald et al., 2014). Similarly, inhibition of cholesterol biosynthesis in the SLE T cells by Atorvastatin reduced signaling and T cell activation (Jury et al., 2006). Statins are widely prescribed as drugs to reduce cholesterol levels by inhibiting 3-hydroxy-3-methylglutaryl-coenzyme-A (HMG-CoA) reductase, which is a key enzyme in the mevalonate pathway to synthesize cholesterol, but also to generate protein prenylations, such as farnesylation or geranylgeranylation. Thus, statins have multiple effects. Indeed, Simvastatin impairs T cell activation through inhibition of Ras prenylation (Ghittoni et al., 2005) and Lovastatin suppresses T cells proliferation due to reduced farnesol pyrophosphate levels (Chakrabarti and Engleman, 1991; Bietz et al., 2017). These anti-inflammatory effects of statins could be beneficial for autoimmune or inflammatory disorders but would worsen immune responses against cancer. In this review, we focus on the cholesterol-related effects.

*In vivo* extraction of cholesterol from plasma membrane of T cells using methyl-β-cyclodextrin in a mouse model of SLE delayed disease onset (Deng and Tsokos, 2008). This is line with reducing the T cells' activity by disruption of TCR nanoclusters and of lipid rafts. The latter mechanism was most likely involved, as clustering of lipid rafts in T cells by cholera toxin B promoted disease progression *in vivo* (Deng and Tsokos, 2008).

To treat cancer by increasing T cell activation has been proven to be a successful strategy (Iwai et al., 2002; Fritz and Lenardo, 2019). The enzyme acyl-CoA cholesterol acyltransferase 1 esterificates cholesterol and thus reduces cholesterol levels in T cells (Chang et al., 2006). Inhibition of this enzyme by Avasimibe led to elevated membrane cholesterol levels in CD8^+^ T cells and enhanced signaling (Yang et al., 2016) most likely by increased TCR nanoclustering. Importantly, this led to enhanced T cell effector functions resulting in stronger killing of tumor cells in mouse melanoma and lung carcinoma models (Yang et al., 2016). Additionally, combination of Avasimibe and anti PD1 treatments proved to be more potent than either monotherapies against cancer (Yang et al., 2016).

These preclinical findings show that the role of cholesterol in promoting TCR signaling (by inducing TCR nanoclustering and formation of lipid rafts) is dominant over its role in suppressing TCR signaling (by stabilizing the *inactive* TCR state).

## Conclusion

Cholesterol specifically binds to the αβ TCR through its TCRβ subunit in the TCR's *inactive* conformation, thus supressing signaling. Cholesterol also promotes TCR nanoclustering and the formation of lipid rafts, both of which promote signaling. In a translational approach, this knowledge was recently used to pharmacologically enhance cholesterol levels in T cells, which potentiated the anti-tumor function of T cells in mouse models. This suggests that the cholesterol-induced nanoclustering and lipid raft formation are dominant in this setting and hence, cholesterol acted as a positive regulator of TCR signaling. What remains to be understood is, how the balance between positive and negative regulation through cholesterol interaction is regulated, in order to achieve fine-tuning of TCR activation and how this can be translated for the treatment of diseases that depends on the sensitivity of TCR activation.

Most likely, the TCR is an example protein for which its regulation by lipids is beginning to unfold. Most likely the influence of direct lipid-TM region interactions on the functioning of membrane proteins is much more widespread than currently thought.

## Author Contributions

SP-C, CD, and WS wrote the first draft of the manuscript and updated the last version. KR, AM, WW, CX, and BO completed and corrected the draft. All authors contributed to the article and approved the submitted version.

## Conflict of Interest

The authors declare that the research was conducted in the absence of any commercial or financial relationships that could be construed as a potential conflict of interest.
